# Anti-HIV antibody development up to 1 year after antiretroviral therapy initiation in acute HIV infection

**DOI:** 10.1172/JCI150937

**Published:** 2022-01-04

**Authors:** Julie L. Mitchell, Justin Pollara, Kenneth Dietze, R. Whitney Edwards, Junsuke Nohara, Kombo F. N’guessan, Michelle Zemil, Supranee Buranapraditkun, Hiroshi Takata, Yifan Li, Roshell Muir, Eugene Kroon, Suteeraporn Pinyakorn, Shalini Jha, Sopark Manasnayakorn, Suthat Chottanapund, Pattarawat Thantiworasit, Peeriya Prueksakaew, Nisakorn Ratnaratorn, Bessara Nuntapinit, Lawrence Fox, Sodsai Tovanabutra, Dominic Paquin-Proulx, Lindsay Wieczorek, Victoria R. Polonis, Frank Maldarelli, Elias K. Haddad, Praphan Phanuphak, Carlo P. Sacdalan, Morgane Rolland, Nittaya Phanuphak, Jintanat Ananworanich, Sandhya Vasan, Guido Ferrari, Lydie Trautmann, on behalf of the RV254 and RV304 Study Groups

**Affiliations:** 1 Vaccine and Gene Therapy Institute, Oregon Health and Science University, Beaverton, Oregon, USA.; 2 US Military HIV Research Program, Walter Reed Army Institute of Research, Silver Spring, Maryland, USA.; 3 Henry M. Jackson Foundation for the Advancement of Military Medicine Inc., Bethesda, Maryland, USA.; 4 Department of Surgery, Duke University Medical Center, Durham, North Carolina, USA.; 5 Department of Medicine and; 6 Center of Excellence in Vaccine Research and Development, Faculty of Medicine, Chulalongkorn University, Bangkok, Thailand.; 7 Department of Medicine, Division of Infectious Diseases and HIV Medicine, Drexel University, Philadelphia, Pennsylvania, USA.; 8 Institute of HIV Research and Innovation (IHRI), Bangkok, Thailand.; 9 Bamrasnaradura Infectious Disease Institute, Nonthaburi, Thailand.; 10 Armed Forces Research Institute of Medical Sciences in Bangkok, Bangkok, Thailand.; 11 Division of AIDS, National Institute of Allergy and Infectious Diseases (NIAID), NIH, Bethesda, Maryland, USA.; 12 HIV Dynamics and Replication Program, National Cancer Institute (NCI), NIH, Frederick, Maryland, USA.; 13 Department of Global Health, University of Amsterdam, Amsterdam, Netherlands.; 14 The RV254 and RV304 Study Groups are detailed in the Supplemental Acknowledgments.

**Keywords:** AIDS/HIV, Immunology, Immunoglobulins

## Abstract

Early initiation of antiretroviral therapy (ART) in acute HIV infection (AHI) is effective at limiting seeding of the HIV viral reservoir, but little is known about how the resultant decreased antigen load affects long-term Ab development after ART. We report here that Env-specific plasma antibody (Ab) levels and Ab-dependent cellular cytotoxicity (ADCC) increased during the first 24 weeks of ART and correlated with Ab levels persisting after 48 weeks of ART. Participants treated in AHI stage 1 had lower Env-specific Ab levels and ADCC activity on ART than did those treated later. Importantly, participants who initiated ART after peak viremia in AHI developed elevated cross-clade ADCC responses that were detectable 1 year after ART initiation, even though clinically undetectable viremia was reached by 24 weeks. These data suggest that there is more germinal center (GC) activity in the later stages of AHI and that Ab development continues in the absence of detectable viremia during the first year of suppressive ART. The development of therapeutic interventions that can enhance earlier development of GCs in AHI and Abs after ART initiation could provide important protection against the viral reservoir that is seeded in individuals treated early in the disease.

## Introduction

Initiation of antiretroviral therapy (ART) during acute HIV infection (AHI) is associated with multiple benefits, including decreased HIV reservoir size (1–6) and preservation of a homogenous viral reservoir with few escape mutations (7). Although it was postulated that the benefits of very early ART initiation would allow for post-treatment control, a treatment interruption study in participants from Thailand who initiated ART in AHI stage 1 reported that all participants experienced viral rebound (8). However, the HIV-specific CD8^+^ T cell response and Env-specific antibody (Ab) titers during viral rebound both correlated negatively with the maximum peak viral load (VL) reached during rebound, suggesting that these responses might partially control post-ART viral replication. We have found that early ART in AHI leads to the preservation of functional immune memory, but the decreased antigen burden also leads to lower numbers of memory T cells after long-term ART (unpublished observations). Similarly, approximately half of the individuals treated in AHI stage 1 either do not develop detectable HIV-specific Abs or serorevert within the first 24 weeks of ART (9). Beyond these limited observations, the impact of early treatment on the development of Abs against HIV has not, to our knowledge, been characterized.

During HIV infection, seroconversion does not occur until the time of peak viremia in stage 3 (S3) of AHI (10, 11). The first free Abs detected in the blood, which recognize gp41 (12), can be cross-reactive with gut flora and show relatively high somatic mutation, indicating that they are probably derived from polyreactive memory B cells activated by the Env protein during the early immune response (13, 14). Abs against gp120 arise later and are initially non-neutralizing (12). Using mathematical modeling, it was determined that the early Ab response to HIV does not significantly affect VL (12), and Ab-selected escape mutations are not identified until the development of autologous neutralizing Abs 3 to 12 months after infection (15–17). However, other Ab functions such as Ab-dependent cellular cytotoxicity (ADCC) and Ab-dependent cellular phagocytosis–mediated (ADCP-mediated) inactivation may potentially provide some protection prior to neutralization (18–21).

In this study, we sought to determine how ART affects the initial development of Env-specific Abs in participants in the Thai RV254 cohort, who initiated treatment in the earliest stages of AHI. We analyzed changes in plasma Env–specific Ab levels, ADCC titers, ADCP scores, and plasma neutralization between the time of ART initiation and initial control of virus during the first 24 weeks of ART. Further, to look at the potential for Ab development after viral suppression, we measured changes in Ab levels and function after a subsequent 24 weeks of clinically suppressed virus.

## Results

**Figure 1:**
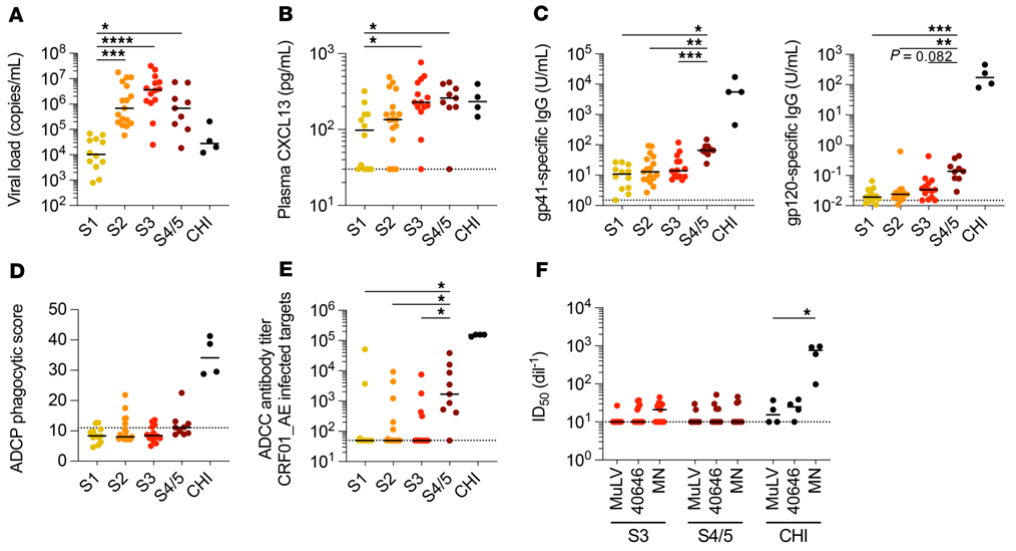
HIV-specific antibodies with ADCC function do not develop until S4/5 of AHI. (**A**) Plasma VL at the time of diagnosis and ART initiation for participants in different stages of AHI or for those with untreated CHI. (**B**) CXCL13 levels in the plasma of participants prior to ART initiation at different stages of AHI or CHI were measured by Luminex assay. (**C**) Levels of plasma gp41-specific and gp120-specific Ab levels were measured by ELISA prior to ART initiation during AHI or CHI. (**D**) ADCP responses of plasma Abs prior to ART initiation during AHI or CHI were measured against the CRF01_AE envelope. The cutoff for positive phagocytosis scores is designated by a dotted line. (**E**) ADCC responses of plasma Abs prior to ART initiation during AHI or CHI were measured using target cells infected with CRF01_AE virus. (**F**) Plasma neutralization was measured in TZM.bl cells using PSVs containing HIV 40646v01 (CRF01_AE), MN.3 (clade B), or MuLV (negative control). The plasma dilution necessary to achieve 50% neutralization (ID_50_) is shown for plasma collected prior to ART initiation in AHI or CHI. Differences were measured by a Kruskal-Wallis test with Dunn’s multiple-comparison test of AHI stages. *n =* 12 for S1; *n =* 17 for S2; *n =* 14 for S3; and *n =* 9 for S4/5. *P < 0.05, **P < 0.01, ***P < 0.001, and ****P < 0.0001.

To characterize the effects of ART administration in AHI on the development of Ab responses directed against HIV, we measured plasma Ab levels and ADCC titers in 52 participants of the RV254 Thai cohort, who were diagnosed with AHI and immediately started on ART (22). All participants were males of similar age, who were infected mostly with the CRF01_AE HIV clade, and the stage of infection was classified using the fourth-generation HIV test (refs. 10, 11 and Table 1). With this staging, the median time to S1 is 2 days shorter than for Fiebig I, resulting in lower median VLs in S1, and S2 is reached approximately 5 days later (11). Classification for S3 and later is equivalent to Fiebig staging, with S3 being coincident with the peak VL (median of 13 days after initial virus detection) and S4/5 encompassing the time span of virus decline and establishment of the set-point VL (10, 23). Participants treated in S1 of AHI had significantly lower VLs at diagnosis than did the other participants (*P <* 0.001 vs. S2, *P <* 0.0001 vs. S3, *P <* 0.05 vs. S4/5; Table 1 and Figure 1A). For reference, we measured Ab levels and ADCC responses in plasma samples from participants in the RV304 Thai cohort who had untreated (*n =* 4) or treated (*n =* 8) chronic HIV infection (CHI).

B cell activation, somatic hypermutation, and class-switching occur within germinal centers (GCs) of secondary lymphoid organs during the development of humoral responses (24). Plasma CXCL13 levels, which can be used as a marker of GC activity (25), were measured at enrollment and were higher with later stages of infection at diagnosis (*P <* 0.05 S1 vs. S3 and S4/5; Figure 1B). We observed similar kinetics for the magnitude of plasma gp41–specific and gp120–specific Ab responses, both of which were significantly higher in participants in S4/5 than in those in earlier stages of AHI (*P <* 0.05), albeit much lower than in participants with CHI (Figure 1C). The quality of the Ab response against HIV, rather than the Ab quantity, has been associated with better viral control in elite controllers and with vaccine protection in the RV144 trial in Thailand (26–29). Very few participants had positive ADCP scores in AHI (Figure 1D), but participants in S4/5 had significantly higher ADCC titers against CRF01_AE-infected targets than did those in earlier stages of AHI (*P <* 0.05; Figure 1E). Finally, as neutralizing antibodies do not arise until 3 to 12 months after HIV infection, we measured only low-titer, nonspecific inhibition of HIV strains in plasma from participants in AHI (Figure 1F). Together, these data indicate that, despite the fact that HIV DNA is measurable in the lymph nodes as early as AHI S1 (1), measurable GC activity and Ab development were delayed until after peak viremia in AHI.

### Virus suppression after ART initiation.

**Figure 2:**
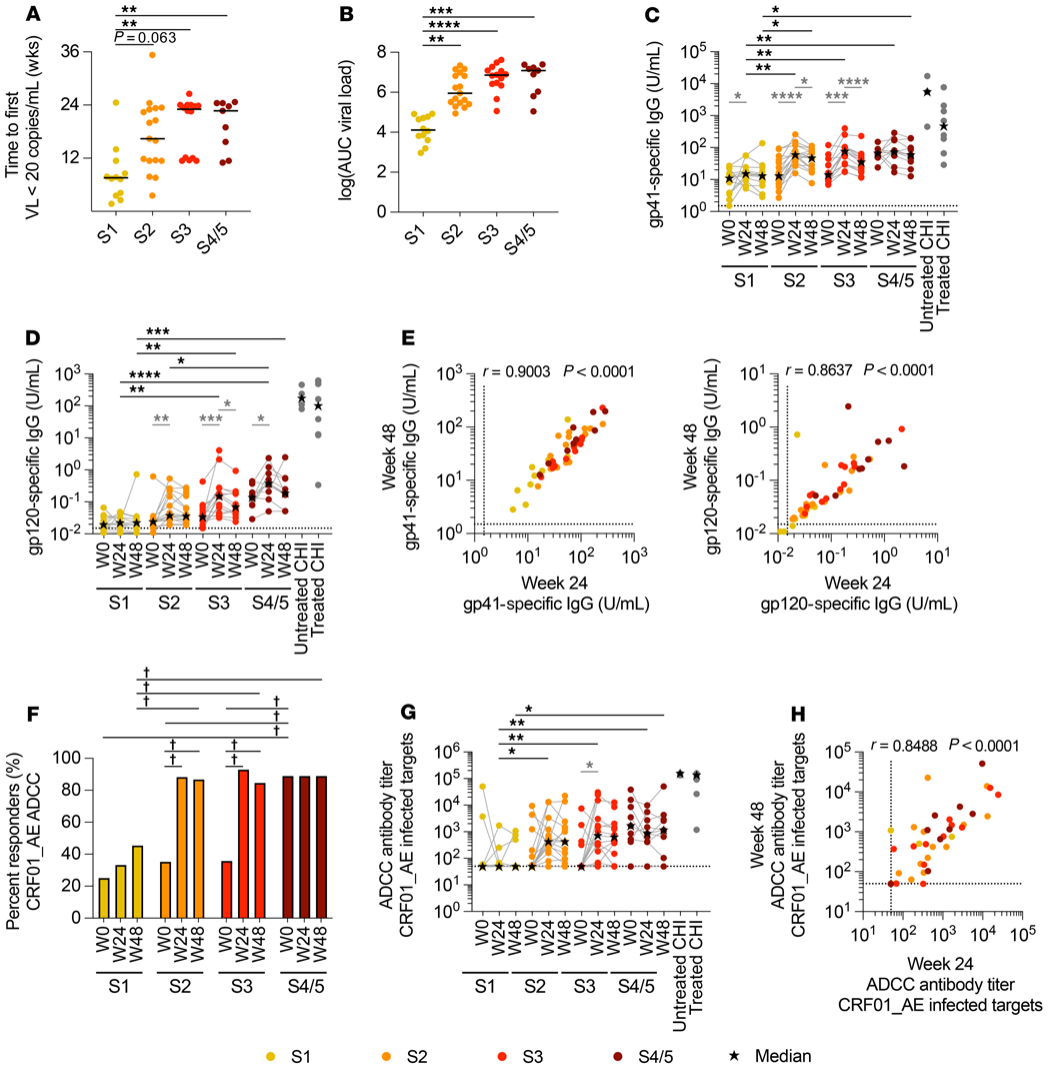
Continued Ab development after ART initiation in participants treated in S2 or later of AHI. (**A**) Time required for the VL to first reach undetectable levels (VL 20 copies/mL) after ART initiation for participants who initiated ART at different stages of AHI. (**B**) The log VL AUC was calculated for each participant. Plasma levels of gp41-specific (**C**) and gp120-specific (**D**) Abs were measured by ELISA after 24 (W24) and 48 (W48) weeks of ART. (**E**) Correlations between Ab levels at week 24 and week 48 are shown for gp41-specific and gp120-specific Abs. (**F**) Proportion of participants who had measurable ADCC responses (responders) at each visit using target cells infected with CRF01_AE virus. (**G**) ADCC function of plasma Abs after 24 and 48 weeks of ART in participants who initiated treatment during AHI or CHI. (**H**) Correlation between ADCC Ab titers at week 24 and week 48. Differences in the proportion of participants responding at each visit were calculated using a χ^2^ test and the Marascuilo procedure (dagger symbol indicates significant differences in proportions). Differences in VLs, AUC, or Ab levels between AHI stages were measured by a Kruskal-Wallis test with Dunn’s multiple-comparison test (black asterisks). Differences in Ab levels between visits were measured by a Wilcoxon matched-pairs, signed-rank test (light gray asterisks). For week 0 and week 24: *n =* 12 for S1; *n =* 17 for S2; *n =* 14 for S3; and *n =* 9 for S4/5. For week 48: *n =* 11 for S1; *n =* 15 for S2; *n =* 13 for S3; and *n =* 9 for S4/5. *P < 0.05, **P < 0.01, ***P < 0.001, and ****P < 0.0001

It is clear that ART initiation has potent effects on the initial reduction of virus replication, but complete viral suppression can take months. As expected, given their lower VL at ART initiation (Figure 1A), the participants treated in S1 of infection had a significantly shorter time to first undetectable VL (≤20 copies/mL) than did those treated in S3 and S4/5 of AHI (*P <* 0.01; Figure 2A). By the 24-week visit, all but 1 participant had an undetectable VL (≤20 copies/mL), and all participants had a VL of ≤20 copies/mL at 36 and 48 weeks. To estimate the antigen load for each participant, we calculated the VL AUC, which was defined as the sum of the post-diagnosis AUC and an imputed AUC that was modeled on data from the RV217 untreated acute infection cohort and accounts for the level of viremia before diagnosis (23, 30). We found that there was no significant difference in the VL AUC between participants who initiated treatment in S2, S3, and S4/5 of AHI (Figure 2B and Supplemental Figure 1A). Analysis of single-copy VLs revealed that the majority (84%) of participants who initiated treatment in S2 of AHI or later had detectable VLs in week 24 using the ultrasensitive assay, but these levels decreased between week 24 and week 48 (S2 and S4/5: *P <* 0.05; 56% detectable; Supplemental Figure 1B). Together, these data indicate that participants who initiated treatment in S3 and S4/5 of AHI had similarly higher antigen exposure during AHI and early ART, but after 24 weeks of ART, there was almost no antigen present in the blood (S3: 1.6 copies/mL at week 24 and 0.63 copies/mL at week 48; S4/5: 0.76 copies/mL at week 24 and 0.20 copies/mL at week 48).

### Env-specific Ab levels increase during the first 6 months of ART.

To determine whether Ab development occurs after ART initiation, we analyzed Ab levels and function after 24 and 48 weeks of ART. Participants treated in S1, S2, and S3 of AHI had elevated gp41-specific Abs at week 24 compared with levels at enrollment (*P <* 0.05), but those treated in S1 had significantly lower Ab levels in weeks 24 and 48 than did participants treated in later stages of AHI (*P <* 0.05; Figure 2C); gp41-specific Ab levels were lower in participants treated in AHI than in those treated during CHI. For gp120-specific Abs, participants treated in both S1 and S2 had lower levels than did those treated at later stages (S1: *P <* 0.0001, S2: *P <* 0.05 vs. S4/5), even though participants treated in S2 had an increase in gp120-specific Ab levels at week 24 (*P <* 0.01; Figure 2D). Both gp41- and gp120-specific Ab levels strongly correlated between weeks 24 and 48 (gp41: *r* = 0.90, *P <* 0.0001; gp120: *r* = 0.86, *P <* 0.0001; Figure 2E), even though participants tended to have lower Ab levels in week 48 compared with week 24 (Figure 2, C and D).

Phagocytic scores were elevated at week 24 for participants who were treated in S3 (*P <* 0.01) and S4/5 (*P =* 0.13; Supplemental Figure 1C), although there was no significant increase in the number of participants with positive phagocytic scores (scores >11). A significantly larger number of participants treated in S2 and S3 of AHI had measurable CRF01_AE-specific ADCC responses after 24 weeks of ART than at the time of ART initiation (Figure 2F). Consistent with low Env-specific Ab levels, less than 50% of the participants treated in S1 developed measurable ADCC responses (Figure 2F). In contrast, despite lower levels of gp120-specific Ab levels in S2 compared with S3 and S4/5, we observed no difference in the average potency of the CRF01_AE-specific ADCC responses (Figure 2G). As observed with the gp41-specific and gp120-specific Abs, we found a strong correlation between the CRF01_AE-specific ADCP and ADCC responses at weeks 24 and 48 of ART (*r* = 0.83 and *r* = 0.85, respectively, *P <* 0.0001; Figure 2H and Supplemental Figure 1D). To determine whether the increased Ab functionality was due to changes in Ab binding, we measured Ab binding to CRF01_AE-infected cells by flow cytometry. Similar to increases in gp120-specific Ab levels, Ab binding increased between week 0 and week 24 in participants treated in S2 or later (*P <* 0.05) and tended to decline by week 48 (Supplemental Figure 1E). The level of Ab binding at week 48 correlated well with gp120-specific Ab levels, and weakly with phagocytic scores and ADCC responses (Supplemental Figure 1F).

In contrast to increases in ADCP and ADCC activity, no increase in neutralization was detected after early ART initiation (Supplemental Figure 1G). Plasma neutralization was measured in participants who initiated treatment in S3 or later using pseudoviruses (PSVs) produced with an ART-resistant backbone to minimize viral inhibition mediated by plasma ART (Supplemental Figure 1H). We detected low-titer, nonspecific inhibition of 2 HIV strains (MN.3 and 40646v01) and MuLV (negative control) that was likely due to plasma ART rather than HIV-specific Abs.

**Figure 3:**
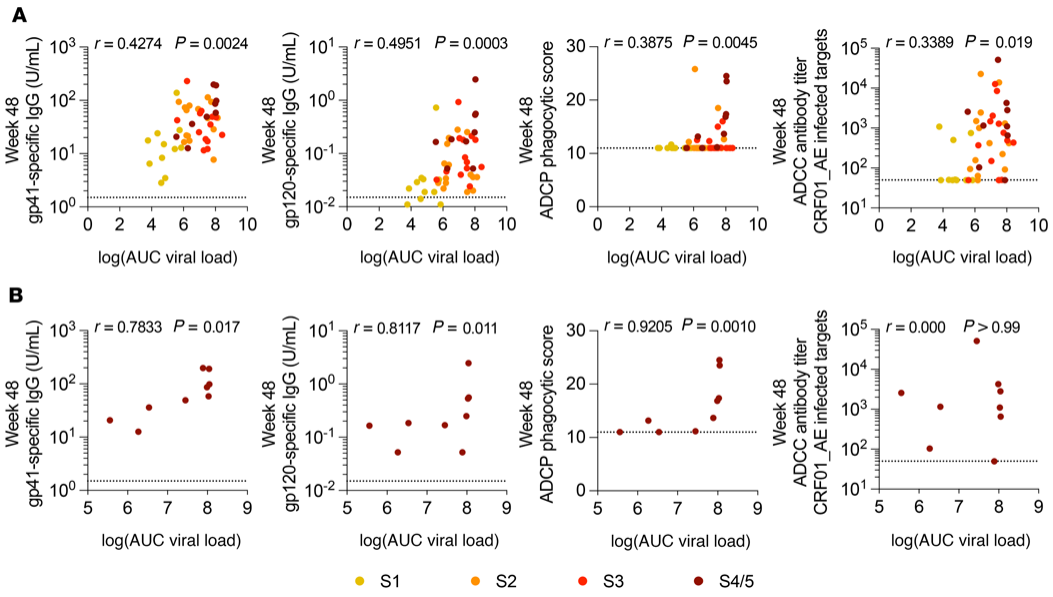
Env-specific Ab levels correlate with the VL AUC only in participants treated in S4/5 of AHI. Correlations between the VL AUC and levels of gp41-specific Abs, gp120-specific Abs, ADCP, and CRF01_AE-specific ADCC titers at week 48 after ART initiation in all participants (**A**) and only those who initiated ART in S4/5 of AHI (**B**). Correlations were measured by Spearman’s correlation. *n =* 11 for S1; *n =* 15 for S2; *n =* 13 for S3; and *n =* 9 for S4/5.

To determine how antigen levels affected the development of the Ab response after ART, we analyzed correlations with VL measures. We identified weak correlations between single-copy VL and Env-specific Ab levels after 24 and 48 weeks of ART (Supplemental Figure 2), suggesting that ongoing low-level viremia does not significantly contribute to Ab levels during early ART. Moreover, when considering participants treated in all stages of AHI, we noted only modest correlations between the VL AUC and all Ab measures at week 48 that were driven by the AHI stage at ART initiation (Figure 3A). When analyzed at the level of the individual stages, we found that the VL AUC had a significant correlation with Env-specific Ab levels and ADCP, but not ADCC, only in participants who initiated ART in S4/5 of AHI (gp41: *r* = 0.78, *P <* 0.05; gp120: *r* = 0.81, *P <* 0.05; ADCP: *r* = 0.92, *P <* 0.01; Figure 3B and Supplemental Figure 3, A–C). Interestingly, after 48 weeks of ART, the levels of Ab binding also correlated with gp120-specific Ab levels and ADCP, but not ADCC (Supplemental Figure 3D). Our data suggest that the viral antigen load after ART (VL AUC) may be associated with the development of HIV-specific binding Ab levels but not ADCC only in participants treated in S4/5 of AHI, and that other factors must contribute to the development of ADCC responses in participants who initiate treatment during AHI.

### Development of increased cross-clade Ab responses.

**Figure 4:**
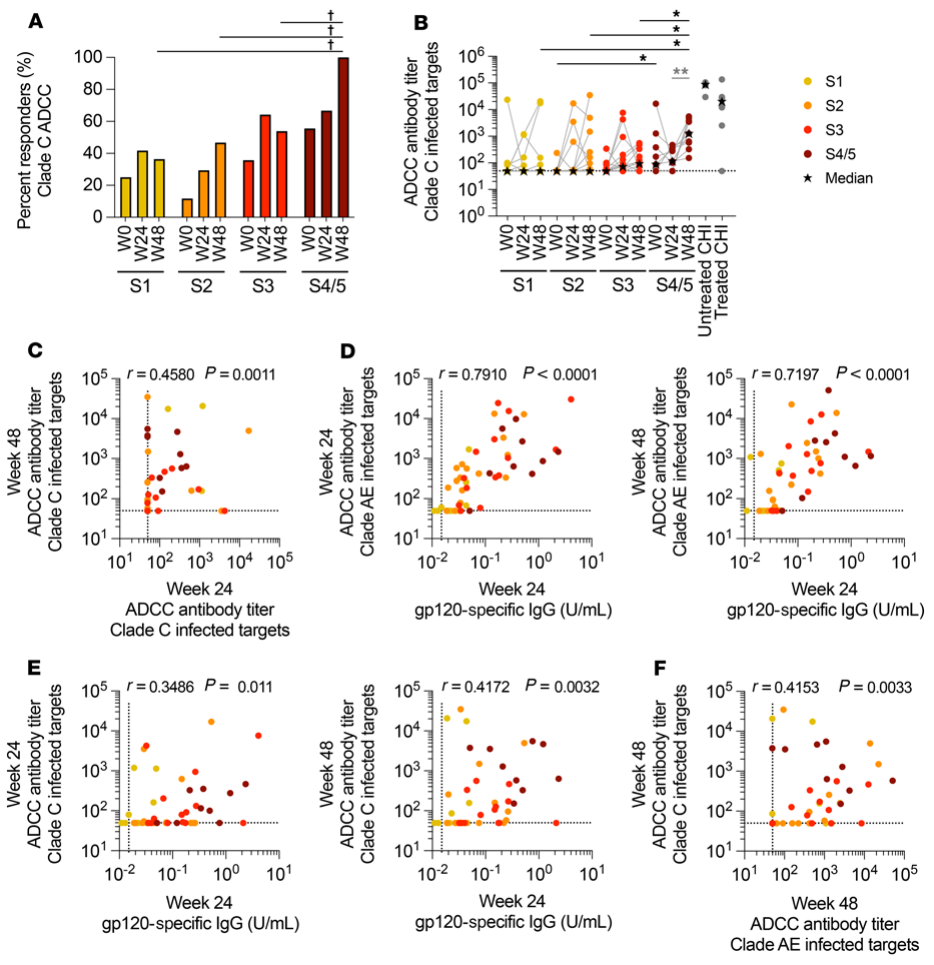
Increased cross-clade ADCC Ab responses between 24 and 48 weeks of ART in participants treated in S4/5 of AHI. Cross-clade ADCC responses were measured in the plasma at week 0, week 24, and week 48 after ART initiation in participants who initiated treatment in AHI or CHI using target cells infected with clade C virus. (**A**) The frequency of participants who had measurable clade C–specific ADCC responses (responders) within each group is shown for each visit. (**B**) Changes in clade C–specific ADCC Ab titers at each visit are shown, with stars representing median titers. (**C**) Correlation between clade C–specific ADCC Ab titers at week 24 and week 48. (**D** and **E**) Correlations between gp120-specific Ab levels at week 24 and CRF01_AE- (**D**) or clade C–specific (**E**) ADCC Ab titers at week 24 and week 48. (**F**) Correlation between CRF01_AE- and clade C–specific Ab titers at week 48. Differences in the proportion of participants who showed a response at each visit were calculated using a χ^2^ test and the Marascuilo procedure (dagger symbol in **A** indicates significant difference in proportions). Differences between AHI stages were measured by a Kruskal-Wallis test with Dunn’s multiple-comparison test (black asterisks in **B**). Differences in ADCC Ab titers between visits were measured by a Wilcoxon matched-pairs, signed-rank test (light gray asterisks in **B**). Correlations were measured by Spearman’s correlation. For week 0 and week 24: *n =* 12 for S1; *n =* 17 for S2; *n =* 14 for S3; and *n =* 9 for S4/5. For week 48: *n =* 11 for S1; *n =* 15 for S2; *n =* 13 for S3; and *n =* 9 for S4/5. *P < 0.05 and **P < 0.01.

To further understand how the Ab response develops after early ART initiation, we measured cross-clade ADCC responses. As participants in this study were infected with CRF01_AE and AE/B recombinant viruses, we measured cross-clade activity against targets infected with the clade C strain TV1, which has recently been used in the HVTN 702 vaccine study and clusters separately from the CRF01_AE CM235 virus (31). We found that few participants developed cross-clade ADCC responses during the early stages of AHI, but over 50% of participants in S4/5 had clade C–specific ADCC responses (Figure 4A). After 24 weeks on ART, an increased proportion of participants who initiated treatment in S3 had detectable clade C–specific ADCC responses (64%, *P =* 0.13). Intriguingly, although there was little change in the magnitude of CRF01_AE-specific ADCC responses between weeks 24 and 48 (Figure 2F), participants treated in S4/5 of AHI had significant increases in the magnitude of clade C–specific ADCC responses after 48 weeks of ART (Figure 4B). Despite having similar times to viral suppression and VL AUC (Figure 2, A and B), participants treated in S4/5 of AHI had higher cross-clade ADCC titers after 48 weeks of ART than did those treated earlier in AHI (*P <* 0.05), with all participants treated in S4/5 developing measurable cross-clade ADCC potency (Figure 4, A and B). We found a trend toward a weak correlation between the VL AUC and cross-clade ADCC responses at week 48 of ART (*r* = 0.21, *P =* 0.16) and a trend toward a modest correlation for participants treated in S4/5 of AHI when stratified by stage (*r* = 0.57, *P =* 0.12; Supplemental Figure 4, A and B). We also noted a correlation between the clade C–specific ADCC responses at weeks 24 and 48 (*r* = 0.46, *P <* 0.01; Figure 4C), but it was weaker than the correlations seen for other Ab measures (Figure 2, E and H and Supplemental Figure 5). Further, while the levels of gp120-specific Abs at week 24 correlated moderately with CRF01_AE-specific ADCC responses (week 48: *r* = 0.72, *P <* 0.0001), the correlations with clade C–specific ADCC responses were weaker (week 48: *r* = 0.42, *P <* 0.01; Figure 4, D and E). Likewise, there was only a modest correlation between the CRF01_AE-specific and clade C–specific ADCC responses at week 48 (*r* = 0.42, *P <* 0.01) (Figure 4F). Thus, the development of cross-clade antibodies was only weakly correlated with the autologous gp120 and ADCC responses. Last, increased Ab binding to clade C–infected cells was not detected after ART, nor did Ab binding correlate with ADCC Ab titer (Supplemental Figure 4, C and D). Together, these data suggest that participants who initiated treatment in S4/5 of AHI had a greater capacity to develop cross-clade ADCC antibodies from week 24 to week 48 of suppressive therapy.

## Discussion

Here, we describe for the first time to our knowledge the development of cross-clade ADCC responses after viral suppression with early ART initiation specifically in participants treated in S4/5, suggesting a more developed GC response capable of supporting Ab evolution after peak viremia in AHI. Indeed, it was in S4/5 that we measured increased levels of Env-specific Abs, ADCC potency, and plasma CXCL13 levels during AHI. In addition to more developed GCs, the advanced Ab development in participants treated in S4/5 after viral suppression in the blood may have been a result of increased virus trapping by follicular dendritic cells (FDCs) in the GCs. HIV immune complexes (ICs) are trapped and retained by FDCs in an infectious state for months for continued selection of high-affinity B cells after viral control (32–38). Indeed, ongoing Ab maturation has been reported to occur over months after flu vaccination and SARS-CoV2 infection (39, 40). Deposition of virus on the FDC network is not evident until 2 weeks after SIV infection (41, 42), and minimal virus deposition has been shown as early as 1 month after symptoms in AHI (43), but the kinetics of HIV trapping and retention by FDCs in the earliest stages of AHI in humans, especially in the context of early ART initiation, is not known. However, the elevated levels of HIV-specific Abs found in participants in S4/5 of AHI could allow for greater IC formation and thus HIV trapping by FDCs, providing for more prolonged antigen exposure in the GC and enhanced Ab development in these participants after ART. As very low levels of virus have been detected on the FDC network even after 26 weeks of ART in SIV-infected nonhuman primates by RNAscope (44), it is possible that virus trapping on the FDC network could be measured in future studies that include the collection of lymph node biopsies or in nonhuman primate studies to investigate whether virus retention in the GC promotes Ab development after early ART initiation.

This study highlights the distinctions in Ab development in participants treated in different stages of AHI. Participants who were treated in S1 of AHI had lower gp41-specific Ab levels, and those who were treated in S1 and S2 of AHI had lower levels of gp120-specific Ab levels than did individuals treated later in AHI. It has been shown that gp41-specific Ab responses arise earlier in AHI and have cross-reactivity with commensal bacteria in the gut, whereas the gp120-specific responses take longer to develop (12–14). These data suggest that participants who initiated ART in S1 and S2 of AHI did not have a sufficient antigen load, duration of antigen exposure, or GC activity to induce significant de novo gp120-specific Ab production during the period of viral suppression after ART initiation. Delaying ART initiation for the sake of Ab development is not advised because of the concomitant seeding of the viral reservoir, but identification of interventions that can be administered at ART initiation to stimulate GC development and promote Ab maturation in individuals who initiate treatment early in AHI could help provide better protection after treatment interruption.

As this study was done in a relatively homogenous group of participants, further work will be needed to determine whether similar Ab kinetics occur in women, individuals of other ethnicities or different ages, and individuals infected with other HIV clades. We focused our analysis on Ab function, as isolation of HIV-specific memory B cells is likely to be challenging given their extremely low frequency in early-treated individuals. However, analysis of Env-specific B cell clones would clarify whether there is continued somatic hypermutation after ART and may be possible in nonhuman primate studies or individuals treated at a later stage of infection. We measured ADCC activity because of the correlations with protection found in the RV144 trial that was completed in Thailand. Although a similar protection was not observed in the corresponding vaccine trial in South Africa, a recent study also found that Ab-mediated NK cell activation was associated with delayed rebound after treatment interruption in participants in Thailand (45), suggesting an important role of ADCC function in HIV control, at least in the context of HIV infection in Thailand. We also measured plasma neutralization and ADCP, which has been associated with a reduced risk of HIV acquisition in clinical and preclinical studies (20, 21), but found little to no activity in these early-treated participants. Although we did not measure either Ab-dependent complement deposition, which is mediated in early AHI by IgM antibodies that wain after ART initiation, or Ab-dependent trogocytosis, which correlates with ADCP activity, these may be of interest in future studies.

Our study provides insights into Ab development in individuals treated in the early stages of infection and the development of cross-clade Ab responses during ART. Participants treated in S2 or later of AHI had a sufficiently large antigen load in the lymph nodes to develop CRF01_AE-specific Abs in the first 24 weeks of ART, whereas those treated in S1 did not. But only those treated in S4/5 showed increased cross-clade ADCC potency after 48 weeks of ART, suggesting that GC development was more advanced in these individuals. However, the GC activity and antigen load were not sufficient to foster significant development of neutralizing Ab responses in these participants, and, as such, all individuals who start treatment in AHI may benefit from concurrent therapeutic vaccination or other interventions that target GC development and Ab maturation when ART is initiated. It is unknown whether continued development of Ab depth and breadth after ART would be seen in participants treated during chronic infection. Individuals in chronic infection have higher Ab levels and ADCC responses before ART and also have altered B cell phenotypes (46) and increased collagen deposition in lymph nodes (47), which could affect further Ab development after ART. However, the prospect of continued Ab development after ART initiation, even in chronic infection, is intriguing, as recent studies suggest that the HIV reservoir is dominated by viruses that are circulating at the time of ART initiation (48, 49). Thus, maturation of the Ab response against the HIV reservoir at the time of ART initiation could have important implications for HIV remission studies and is in line with recent data showing that autologous IgG antibodies block the outgrowth of a substantial but variable fraction of viruses in the latent reservoir for HIV-1 (50). Thus, any Ab development after ART initiation could produce better antibodies to control the virus after treatment interruption and is worth further study.

## Methods

### Study participants.

Plasma samples from 64 participants were analyzed to determine HIV-specific Ab development in AHI and after early ART initiation. Fifty-two of the participants were enrolled in the RV254/SEARCH 010 cohort at the Thai Red Cross AIDS Research Centre in Bangkok, Thailand (clinicaltrials.gov identifier NCT00796146; ref. 4). This study enrolled individuals who were diagnosed with HIV in the earliest stages of acute infection and who were offered ART as part of a separate protocol (NCT00796263). For the purpose of the current study, participants were categorized into stages 1–5 of AHI on the basis of previously reported staging strategies (refs. 10, 11 and Table 1): S1, positive HIV RNA, nonreactive fourth generation (4G) immunoassay (IA), nonreactive 3G IA; S2, positive HIV RNA, reactive 4G IA, nonreactive 3G IA; S3, positive HIV RNA, reactive 4G IA, reactive 3G IA, negative Western blot (WB); S4, positive HIV RNA, reactive 4G IA, reactive 3G IA, intermediate WB; S5, positive HIV RNA, reactive 4G IA, reactive 3G IA, positive WB except for p31. Longitudinal samples from the time of enrollment (week 0), the 24-week visit, and the 48-week visit were analyzed for all participants, as available. Samples from participants with untreated CHI (*n =* 4) and treated CHI (*n =* 8; median of 44 months on ART, IQR: 25–116 months) from the RV304/SEARCH013 cohort (NCT01397669) were also analyzed.

### Measurement of HIV-1 RNA.

Plasma VL was measured quantitatively with the Roche Amplicor version 1.5 ultrasensitive assay with a lower limit of quantification of 50 copies/mL (Roche Diagnostics), before replacement with the COBAS TaqMan HIV-1 Test version 2.0 (Roche Diagnostics) with a lower limit of quantification of 20 copies/mL. Plasma VL was measured at the time of study enrollment, every 2 weeks from the date of enrollment through week 4 of ART, every 4 weeks from week 4 through week 24 of ART, and every 12 weeks thereafter. Single-copy HIV-1 RNA levels were measured retrospectively using ultrasensitive hybrid real-time/digital PCR, as previously described (51, 52). The AUC was calculated by adding the AUC measured in the study starting at ART initiation and the estimated AUC at each AHI stage derived from the VL trendline in untreated AHI (23, 30).

### Measurement of plasma CXCL13 levels.

Plasma CXCL13 levels were measured using Luminex technology with a ProcartaPlex Multiplex Immunoassay (Assay MXH49YW, Life Technologies, Thermo Fisher Scientific). Samples were run according to the manufacturer’s instructions, and cytokine standards were provided by the manufacturer. A Bio-Plex 200 system was used to acquire samples, and the data were analyzed with Bio-Plex Manager Software (both from Bio-Rad).

### Measurement of HIV-specific Ab levels.

HIV-specific Ab levels were measured as previously described (53, 54). High-binding, half-area microplates (Grenier Bio-One) were coated with recombinant gp41 (HIV-1 Envelope, ProSpec) or gp120 consensus CRF01_AE (Immune Technology Corp.) protein at a concentration of 1 μg/mL in PBS overnight at 4°C. The next day, the plates were washed 5 times (unless otherwise stated) with wash buffer (PBS plus 0.05% Tween 20) and then blocked for 1 hour with PBS plus 10% (vol/vol) FBS at room temperature (RT). The plates were washed again before plasma and standards were added in duplicate at different dilutions and incubated for 2 hours at RT. For standard curves, 2-fold dilutions of anti-gp120/anti-gp160 (CRF01_AE) (Immune Technology Corp., clone 26A4) or human HIV immunoglobulin (NIH AIDS Research and Reference Program, Division of AIDS, NIAID, NIH; provided by Elias Haddad, Drexel University, Philadelphia, Pennsylvania, USA) were used. After subsequent washing, the plates were incubated for 1 hour at RT with 1 μg/mL biotin-conjugated anti–human IgG (Mabtech, clone MT78/145) or anti–mouse IgG (Mabtech) for the gp120 standard curve. The plates were then washed and incubated with streptavidin-HRP (Mabtech) for 1 hour at RT. After a final wash, 50 μL 3,3′,5,5′-tetramethylbenzidine (TMB) substrate (MilliporeSigma) was added until the appearance of color, and the enzymatic reaction was stopped by adding 50 μL of 1M H_3_PO_4_. The absorbance was read at 450 nm using a Versamax Tunable Microplate Reader (Molecular Devices). Absorbance values were converted to concentrations using SoftMax Pro Software (Molecular Devices) to calculate a 4-parameter logistic fit of the standard curve. The mean concentration of duplicate wells is reported as U/mL, with the gp120 standard curve measured as ng/mL anti-gp120/anti-gp160 Abs and the gp41 standard curve as g/mL total IgG in the HIV immunoglobulin standard.

### Infectious molecular clones.

The HIV-1 reporter viruses used were replication-competent infectious molecular clones (IMCs) designed to encode the *env* genes of CM235 (subtype A/E; GenBank no. AF259954.1) and TV-1 (subtype C; GeneBank No. HM215437) as previously described (55).

### Infection of the CEM.NKR_CCR5_ cell line with HIV-1 IMCs.

CEM.NKR_CCR5_ cells were infected with HIV-1 IMCs as previously described (55). Briefly, IMCs were titrated in order to achieve maximum infection within 48–72 hours of infection, as determined by the detection of luciferase activity and intracellular p24 expression. For each ADCC assay, the frequency of infected target cells was monitored by intracellular p24 staining. Assays performed using infected target cells were considered reliable if cell viability was 60% or greater, and the percentage of viable p24^+^ target cells on assay day was 20% or greater.

### Luciferase ADCC assay.

We used a modified version of the ADCC luciferase assay (56). Briefly, CEM.NKR_CCR5_ cells were infected with HIV-1 IMCs as described above and used as target cells. For effector cells, cryopreserved PBMCs obtained by leukapheresis from a HIV-seronegative individual (Fc-γ receptor IIIa 158 V/F heterozygous) were thawed the day before the assay and rested overnight in RPMI 1640 medium supplemented with antibiotics and 10% fetal bovine plasma (R10), and with recombinant human IL-15 at a concentration of 10 ng/mL. Effector and target cells (30:1 E/T ratio) were plated in opaque, 96-well half-area plates and cocultured with serial dilutions of plasma. Each plasma sample was assayed at 6 dilutions, starting at a dilution of 1:50, with duplicate wells set up for each dilution. For the 4-fold serial dilution scheme, plasma dilutions of 1:50, 1:200, 1:800, 1:3200, 1:12,800, and 1:51,200 were used. Cocultures were incubated for 6 hours at 37°C in 5% CO_2_ in IL-15–supplemented R10. The assay readout was luminescence intensity (measured in RLU) generated by surviving target cells that had not been lysed by the effector cell population in the presence of ADCC-mediating plasma Abs. The mAb palivizumab (SYNAGIS, Swedish Orphan Biovitrum), which mediates ADCC (57) but is specific for respiratory syncytial virus, and a cocktail of HIV-1 mAbs shown to mediate ADCC (A32 [ref. 58], 2g12 [ref. 59], CH44 [ref. 60], and 7B2 [ref. 61]) were used as negative and positive controls, respectively. All mAbs were generated using the IgG1 constant region containing alanine substitutions (S298A, E333A, K334A) designed to enhance binding to Fc-γ receptor IIIa (FcR3A; ref. 62). A response was reported as positive if the specific killing was greater than 15% at the first 2 dilutions after subtracting the average percentage of specific killing observed by testing a panel of 11 serum samples collected from geographically matched seronegative subjects. The ADCC Ab titer, defined as the last dilution of plasma capable of mediating ADCC in our in vitro assay, was calculated by interpolation of the dilution curve that intersected the positive cutoff of 15% specific killing.

### Ab-binding assay.

Ab binding to HIV-infected cells was measured as described previously (63, 64). Briefly, 7.5 × 10^5^ CM235 or TV1 IMC-infected CEM.NKR_CCR5_ cells were incubated with 100-fold diluted human plasma for 2 hours at 37°C followed by surface staining with anti–IgG-PECy7 secondary Ab (BioLegend, clone M1310G05) and anti–CD4–APC Ab (BioLegend, clone OKT4) for 20 minutes at RT. Cells were then resuspended in 100 μL BD Cytofix/Cytoperm (Thermo Fisher Scientific) and incubated for 20 minutes at 4°C, followed by staining with anti–p24-FITC Ab (Beckman Coulter, clone KC57) for 25 minutes at RT. Cells were washed and resuspended in 125 μL PBS–1% paraformaldehyde. The samples were acquired within 24 hours using a BD Fortessa Cytometer (BD Biosciences), and plasma binding was quantified as the percentage of target cells positive for anti-IgG secondary Ab after background subtraction.

### ADCP assay.

ADCP was measured as previously described (65). Briefly, gp120 CM235 (NIH HIV Reagent Program, Division of AIDS, NIAID, NIH: HIV-1 CM235 gp120 Recombinant Protein, ARP-12816, contributed by NIAID, DAIDS) was biotinylated at a biotin-to-protein ratio of 50 according to the manufacturer’s instructions (Thermo Fisher Scientific) and incubated with yellow-green streptavidin fluorescent beads (Molecular Probes) for 2 hours at 37°C. Ten microliters of a 100-fold dilution of beads-protein was incubated for 2 hours at 37°C with 100 μL of 200-fold diluted plasma samples before addition of Tamm-Horsfall protein 1 (THP-1) cells (20,000/well; MilliporeSigma). After an 18-hour incubation at 37^o^C, the cells were fixed with 4% formaldehyde solution (Tousimis), and fluorescence was evaluated on an LSR II (BD Biosciences). The phagocytic score was calculated by multiplying the percentage of bead-positive cells by the geo MFI of the bead-positive cells and dividing by 10^4^.

### Plasma neutralization assay.

Plasma neutralization was evaluated in a high-throughput TZM.bl PSV assay, as previously described (66). PSVs were produced using an ART-resistant backbone vector (SG3ΔEnv/K101P.Q148H.Y181C; courtesy of Michael S. Seaman, Center for Virology and Vaccine Research, Beth Israel Deaconess Medical Center, Harvard Medical School, Boston, Massachusetts, USA) shown to minimize the background inhibitory activity of antiretroviral drugs present in patients’ plasma (67). PSV Envs included HIV MN.3 (subtype B) and 40646v01 (CRF01_AE) and murine leukemia virus (MuLV) as a nonspecific control. Plasma was diluted 1:10 in growth medium and serially diluted using the Biomek NXP liquid handler (Beckman Coulter). Titered plasma was transferred onto 384-well culture plates and incubated with an equal volume of PSV for 45 minutes at 37°C. TZM-bl cells (3 × 10^3^ cells/well) mixed with DEAE-dextran were added to each well and incubated for an additional 48 hours. RLU were detected with the SpectraMax Paradigm Microplate Reader (Molecular Devices) using the Bright-Glo Luciferase Assay System (Promega). Percentage neutralization (percentage reduction of RLU in the presence of plasma) was calculated for each plasma dilution. Neutralization dose-response curves were fitted by nonlinear regression using the LabKey Server, and the final titer is reported as the reciprocal of the dilution of plasma necessary to achieve 50% neutralization (50% inhibitory dose [ID_50_]).

### Statistics.

Statistical analyses were performed using the Kruskal-Wallis test with Dunn’s multiple-comparison test to measure differences between groups. Comparisons between values at different time points on ART were performed with the Wilcoxon matched-pairs, signed-rank test. Correlations were performed with the nonparametric Spearman test. When multiple comparisons were done for Ab measures within each AHI stage, the Benjamini-Hochberg procedure was used to correct for a FDR of 25%. Differences in the proportion of responders were calculated using a χ^2^ test followed by the Marascuilo procedure. Statistical analyses were performed using GraphPad Prism (GraphPad Software) and R Statistical Software (Foundation for Statistical Computing). Significance was defined as a *P* value of less than 0.05 for 2-sided testing.

### Study approval.

Informed consent was obtained from all participants prior to inclusion in the studies. All studies were approved by the IRBs of Chulalongkorn University and the Walter Reed Army Institute of Research.

## Author contributions

JLM designed the experiments, analyzed the data, and wrote and edited the manuscript. JP, JN, SB, and HT performed the experiments, analyzed data, and edited the manuscript. KD, RWE, KFN, and MZ performed the experiments and analyzed data. RM provided guidance on methodology. EK managed participant recruitment and follow-up and edited the manuscript. SP and SJ provided help with statistical analyses. SM, SC, PT, P Prueksakaew, NR, BN, and CPS managed participant recruitment and follow-up. LF provided study supervision. ST, VRP, and EKH provided resources. DPP and LW designed experiments and edited the manuscript. FM provided single-copy VL data. YL and MR calculated the AUC. P Phanuphak provided support for the clinical studies. NP provided support for the clinical studies and edited the manuscript. JA designed the clinical studies and edited the manuscript. SV provided supervision and edited the manuscript. GF and LT designed the experiments, wrote and edited the manuscript, and provided supervision.

## Supplementary Material

Supplemental data

## Figures and Tables

**Table 1:**
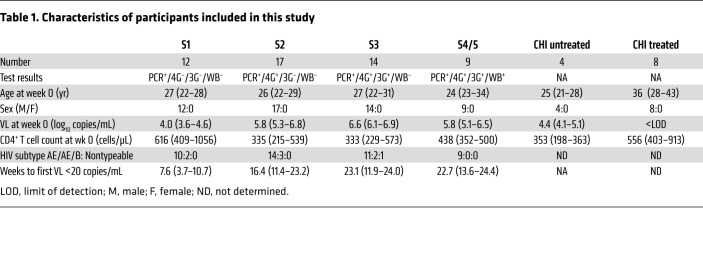
Characteristics of participants included in this study

## Version 1 11/11/2021

In-Press Preview

## Version 2 01/04/2022

Electronic publication

## References

[1] Leyre L (2020). Abundant HIV-infected cells in blood and tissues are rapidly cleared upon ART initiation during acute HIV infection. Sci Transl Med.

[2] Buzon MJ (2014). Long-term antiretroviral treatment initiated at primary HIV-1 infection affects the size, composition, and decay kinetics of the reservoir of HIV-1-infected CD4 T cells. J Virol.

[3] Strain MC (2005). Effect of treatment, during primary infection, on establishment and clearance of cellular reservoirs of HIV-1. J Infect Dis.

[4] Ananworanich J (2012). Impact of multi-targeted antiretroviral treatment on gut T cell depletion and HIV reservoir seeding during acute HIV infection. PLoS One.

[5] Deleage C (2016). Impact of early cART in the gut during acute HIV infection. JCI Insight.

[6] Ananworanich J (2016). HIV DNA set point is rapidly established in acute HIV infection and dramatically reduced by early ART. EBioMedicine.

[7] Lee GQ (2019). HIV-1 DNA sequence diversity and evolution during acute subtype C infection. Nat Commun.

[8] Colby DJ (2018). Rapid HIV RNA rebound after antiretroviral treatment interruption in persons durably suppressed in Fiebig I acute HIV infection. Nat Med.

[9] de Souza MS (2016). Initiation of antiretroviral therapy during acute HIV-1 infection leads to a high rate of nonreactive HIV serology. Clin Infect Dis.

[10] Fiebig EW (2003). Dynamics of HIV viremia and antibody seroconversion in plasma donors: implications for diagnosis and staging of primary HIV infection. AIDS.

[11] Ananworanich J (2013). A novel acute HIV infection staging system based on 4th generation immunoassay. Retrovirology.

[12] Tomaras GD (2008). Initial B-cell responses to transmitted human immunodeficiency virus type 1: virion-binding immunoglobulin M (IgM) and IgG antibodies followed by plasma anti-gp41 antibodies with ineffective control of initial viremia. J Virol.

[13] Liao HX (2011). Initial antibodies binding to HIV-1 gp41 in acutely infected subjects are polyreactive and highly mutated. J Exp Med.

[14] Trama AM (2014). HIV-1 envelope gp41 antibodies can originate from terminal ileum B cells that share cross-reactivity with commensal bacteria. Cell Host Microbe.

[15] Wei X (2003). Antibody neutralization and escape by HIV-1. Nature.

[16] Richman DD (2003). Rapid evolution of the neutralizing antibody response to HIV type 1 infection. Proc Natl Acad Sci U S A.

[17] Gray ES (2007). Neutralizing antibody responses in acute human immunodeficiency virus type 1 subtype C infection. J Virol.

[18] Forthal DN (2001). Antibody from patients with acute human immunodeficiency virus (HIV) infection inhibits primary strains of HIV type 1 in the presence of natural-killer effector cells. J Virol.

[19] Aasa-Chapman MM (2005). Detection of antibody-dependent complement-mediated inactivation of both autologous and heterologous virus in primary human immunodeficiency virus type 1 infection. J Virol.

[20] Neidich SD (2019). Antibody Fc effector functions and IgG3 associate with decreased HIV-1 risk. J Clin Invest.

[21] Om K (2020). Adjuvanted HIV-1 vaccine promotes antibody-dependent phagocytic responses and protects against heterologous SHIV challenge. PLoS Pathog.

[22] Ananworanich J (2016). Virological and immunological characteristics of HIV-infected individuals at the earliest stage of infection. J Virus Erad.

[23] Robb ML (2016). Prospective study of acute HIV-1 infection in adults in East Africa and Thailand. N Engl J Med.

[24] Victora GD, Nussenzweig MC (2012). Germinal centers. Annu Rev Immunol.

[25] Havenar-Daughton C (2016). CXCL13 is a plasma biomarker of germinal center activity. Proc Natl Acad Sci U S A.

[26] Ackerman ME (2016). Polyfunctional HIV-Specific antibody responses are associated with spontaneous HIV control. PLoS Pathog.

[27] Haynes BF (2012). Immune-correlates analysis of an HIV-1 vaccine efficacy trial. N Engl J Med.

[28] Chung AW (2014). Polyfunctional Fc-effector profiles mediated by IgG subclass selection distinguish RV144 and VAX003 vaccines. Sci Transl Med.

[29] Yates NL (2014). Vaccine-induced Env V1-V2 IgG3 correlates with lower HIV-1 infection risk and declines soon after vaccination. Sci Transl Med.

[30] Reeves DB (2021). Timing HIV infection with a simple and accurate population viral dynamics model. J R Soc Interface.

[31] Gray GE (2021). Vaccine efficacy of ALVAC-HIV and bivalent subtype C gp120-MF59 in adults. N Engl J Med.

[32] Nossal GJ (1965). Antigens in immunity. 8. Localization of 125-I-labelled antigens in the secondary response. Immunology.

[33] Chen LL (1978). Anatomy of germinal centers in mouse spleen, with special reference to “follicular dendritic cells”. J Cell Biol.

[34] Aydar Y (2005). The influence of immune complex-bearing follicular dendritic cells on the IgM response, Ig class switching, and production of high affinity IgG. J Immunol.

[35] Heesters BA (2013). Endocytosis and recycling of immune complexes by follicular dendritic cells enhances B cell antigen binding and activation. Immunity.

[36] Smith BA (2001). Persistence of infectious HIV on follicular dendritic cells. J Immunol.

[37] Heesters BA (2015). Follicular dendritic cells retain infectious HIV in cycling endosomes. PLoS Pathog.

[38] Cavert W (1997). Kinetics of response in lymphoid tissues to antiretroviral therapy of HIV-1 infection. Science.

[39] Andrews SF (2019). Activation dynamics and immunoglobulin evolution of pre-existing and newly generated human memory B cell responses to influenza hemagglutinin. Immunity.

[40] Gaebler C (2021). Evolution of antibody immunity to SARS-CoV-2. Nature.

[41] Hurtrel B (1994). Early events in lymph nodes during infection with SIV and FIV. Res Virol.

[42] Canto-Nogues C (2001). In situ hybridization and immunolabelling study of the early replication of simian immunodeficiency virus (SIVmacJ5) in vivo. J Gen Virol.

[43] Pantaleo G (1998). Evolutionary pattern of human immunodeficiency virus (HIV) replication and distribution in lymph nodes following primary infection: implications for antiviral therapy. Nat Med.

[44] Deleage C (2016). Defining HIV and SIV reservoirs in lymphoid tissues. Pathog Immun.

[45] Bartsch YC (2021). Viral rebound kinetics correlate with distinct HIV Antibody Features. mBio.

[46] Austin JW (2019). Overexpression of T-bet in HIV infection is associated with accumulation of B cells outside germinal centers and poor affinity maturation. Sci Transl Med.

[47] Schacker TW (2002). Collagen deposition in HIV-1 infected lymphatic tissues and T cell homeostasis. J Clin Invest.

[48] Abrahams MR (2019). The replication-competent HIV-1 latent reservoir is primarily established near the time of therapy initiation. Sci Transl Med.

[49] Pankau MD (2020). Dynamics of HIV DNA reservoir seeding in a cohort of superinfected Kenyan women. PLoS Pathog.

[50] Bertagnolli LN (2020). Autologous IgG antibodies block outgrowth of a substantial but variable fraction of viruses in the latent reservoir for HIV-1. Proc Natl Acad Sci U S A.

[51] Palmer S (2003). New real-time reverse transcriptase-initiated PCR assay with single-copy sensitivity for human immunodeficiency virus type 1 RNA in plasma. J Clin Microbiol.

[52] Somsouk M (2014). The immunologic effects of mesalamine in treated HIV-infected individuals with incomplete CD4^+^ T cell recovery: a randomized crossover trial. PLoS One.

[53] Cubas RA (2013). Inadequate T follicular cell help impairs B cell immunity during HIV infection. Nat Med.

[54] Muir R (2016). Altered memory circulating T follicular helper-B cell interaction in early acute HIV Infection. PLoS Pathog.

[55] Easterhoff D (2020). Boosting with AIDSVAX B/E enhances env constant region 1 and 2 antibody-dependent cellular cytotoxicity breadth and potency. J Virol.

[56] Pollara J (2014). HIV-1 vaccine-induced C1 and V2 Env-specific antibodies synergize for increased antiviral activities. J Virol.

[57] https://www.criver.com/sites/default/files/resources/MechanismofActionAssaystoDeterminetheFcEffectorFunctionofPalivizumab.pdf.

[58] Ferrari G (2011). An HIV-1 gp120 envelope human monoclonal antibody that recognizes a C1 conformational epitope mediates potent antibody-dependent cellular cytotoxicity (ADCC) activity and defines a common ADCC epitope in human HIV-1 serum. J Virol.

[59] Trkola A (1996). Human monoclonal antibody 2G12 defines a distinctive neutralization epitope on the gp120 glycoprotein of human immunodeficiency virus type 1. J Virol.

[60] Moody MA (2015). Strain-specific V3 and CD4 binding site autologous HIV-1 neutralizing antibodies select neutralization-resistant viruses. Cell Host Microbe.

[61] Mayr LM (2017). Non-neutralizing antibodies targeting the V1V2 Domain of HIV exhibit strong antibody-dependent cell-mediated cytotoxic activity. Sci Rep.

[62] Shields RL (2001). High resolution mapping of the binding site on human IgG1 for Fc gamma RI, Fc gamma RII, Fc gamma RIII, and FcRn and design of IgG1 variants with improved binding to the Fc gamma R. J Biol Chem.

[63] Bradley T (2017). Pentavalent HIV-1 vaccine protects against simian-human immunodeficiency virus challenge. Nat Commun.

[64] Tuyishime M (2020). Improved killing of HIV-infected cells using three neutralizing and non-neutralizing antibodies. J Clin Invest.

[65] Ackerman ME (2011). A robust, high-throughput assay to determine the phagocytic activity of clinical antibody samples. J Immunol Methods.

[66] Wieczorek L (2020). Evaluation of HIV-1 neutralizing and binding antibodies in maternal-infant transmission in Thailand. Virology.

[67] Cohen YZ (2018). Relationship between latent and rebound viruses in a clinical trial of anti-HIV-1 antibody 3BNC117. J Exp Med.

