# Supplementary material for: Anti-HIV antibody development up to 1 year after antiretroviral therapy initiation in acute HIV infection
Source: J Clin Invest. 2022 Jan 4;132(1):e150937. doi: 10.1172/JCI150937 (PMC8718150; doi:10.1172/JCI150937)
Supplement: Supplemental data [file jci-132-150937-s030.pdf]

Supplemental Materials

Supplemental Acknowledgements

The RV254 and RV304 studies include the following team members: Nipat Teeratakulpisarn[1], Supanit Pattanachaiwit[2], Somchai Sriplienchan[1], Ponpen Tantivitayakul[1], Nitiya Chomchey[1], Duanghathai Suttichom[1], Kultida Poltavee[1], Jintana Intasan[1], Tassanee Luekasemsuk[1], Hathairat Savadsuk[1], Somporn Tipsuk[1], Suwanna Puttamsawin[1], Khunthalee Benjapornpong[1], Nisakorn Ratnaratorn[1], Kamonkan Tangnaree[1], Chutharat Munkong[1], Rommanus Thaimanee[1], Patcharin Eamyoung[3], Sasiwimol Ubolyam[3], Sukalya Lerdlum[4], Rungsun Rerknimitr[4], Sunee Sirivichayakul[4], Phandee Wattanaboonyongcharoen[4], Robert O'Connell[5], Alexandra Schuetz[5], Denise Hsu[5], Siriwat Akapirat[5], Nantana Tantibul[5], Nampueng Churikanont[5], Saowanit Getchalarat[5]; Trevor Crowell[6,7], Donn Colby[6,7], Ellen Turk[6,7], Oratai Butterworth[6,7], Mark Milazzo[6,7], Leigh Anne Eller[6,7].

[1] Institute of HIV Research and Innovation (IHRI), Bangkok, Thailand

[2] The Thai Red Cross AIDS Research Centre, Bangkok, Thailand

[3] The HIV Netherlands Australia Thailand Research Collaboration (HIV-NAT), The Thai Red Cross AIDS Research Centre, Bangkok, Thailand

[4] Faculty of Medicine, Chulalongkorn University, Bangkok, Thailand

[5] Armed Forces Research Institute of Medical Sciences in Bangkok, Bangkok, Thailand

[6] U.S. Military HIV Research Program, Walter Reed Army Institute of Research, Silver Spring, MD, USA

[7] Henry M. Jackson Foundation for the Advancement of Military Medicine, Inc., Bethesda, MD, USA

Supplemental Figures

Supplemental Figure 1. Changes in viral load and antibody levels after ART initiation

Supplemental Figure 2. Correlations between single copy viral load and antibody levels.

Supplemental Figure 3. Correlations between AUC viral load and antibody levels.

Supplemental Figure 4. Clade C ADCC antibody titer correlations.

Supplemental Figure 5. Correlations between longitudinal antibody measures by AHI stage when ART was initiated.

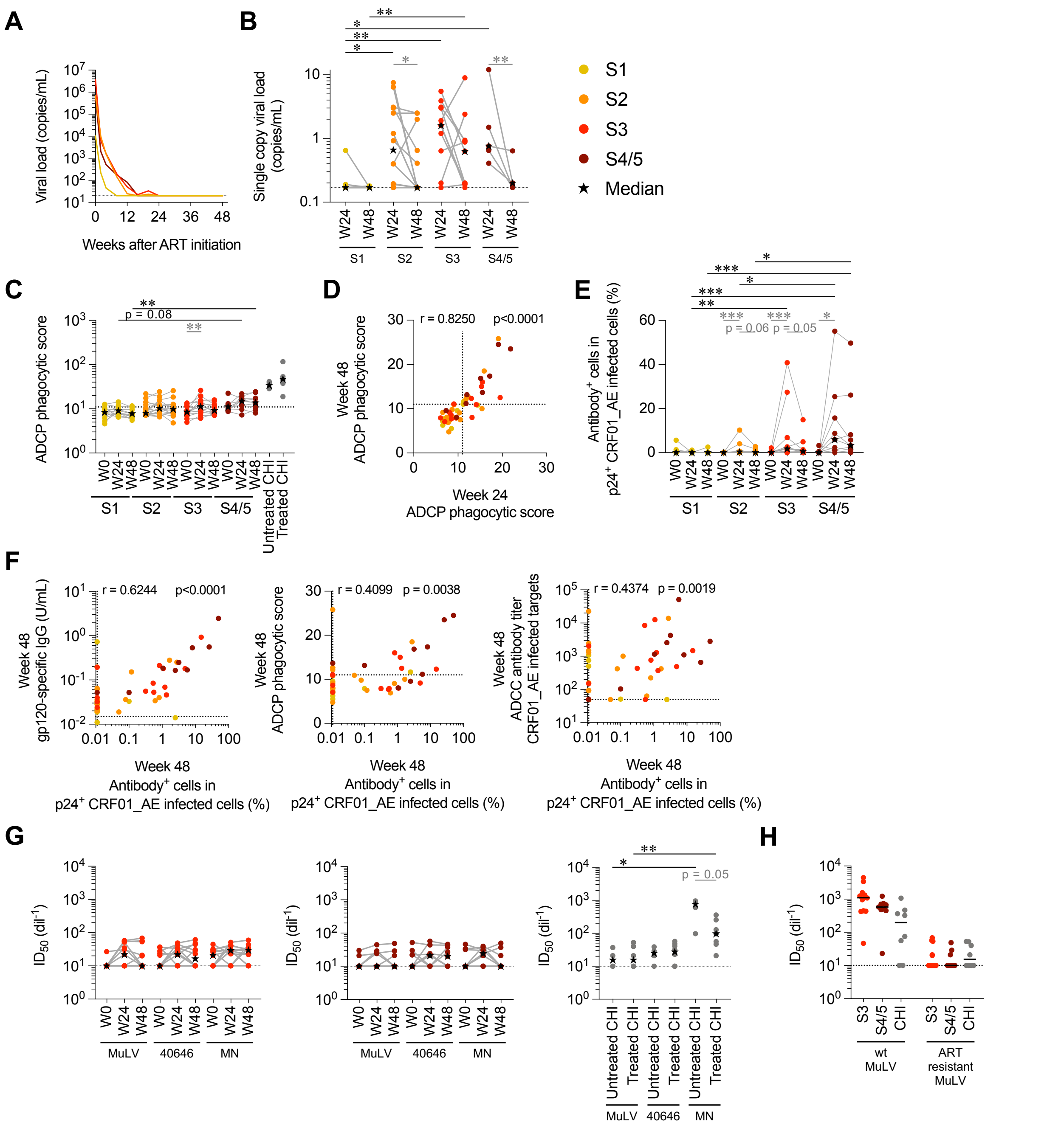

Supplemental Figure 1. Changes in viral load and antibody levels after ART initiation

(A) Median viral loads (VLs) after ART initiation are shown for participants who initiated treatment in the different stages of AHI. **(B)** Single copy HIV-1 RNA levels were measured in the plasma using ultrasensitive PCR at 24 and 48 weeks of ART, and changes between the two visits are shown. **(C)** ADCP was measured at ART initiation or after 24 and 48 weeks of ART in

participants who initiated treatment in the different stages of AHI or in CHI. **(D)** Correlation between ADCP phagocytic scores at week 24 and week 48 of ART. **(E)** Antibody binding activity was measured using plasma collected at ART initiation or after 24 and 48 weeks of ART. The percent of $p24^{+}$ CRF01_AE infected cells stained with anti-IgG secondary antibody after subtraction of background is shown. **(F)** Correlations between the level of antibody binding activity and gp120-specific antibody levels, ADCP phagocytic score, and ADCC antibody titers after 48 weeks of ART. **(G)** Plasma neutralization was measured in participants who initiated ART in S3 and S4/5 of AHI or in CHI using pseudoviruses produced with an ART-resistant vector background. The ID_{50} is shown for pseudoviruses containing HIV 40646v01 (CRF01_AE), MN.3 (Clade B), or murine leukemia virus (MuLV; negative control). **(H)** Comparison of viral neutralization of MuLV expressed by pseudovirus produced with either the wild-type (wt) or ART-resistant vector by plasma collected after 48 weeks of ART. Differences between AHI stages were measured by a Kruskal-Wallis test with Dunn's multiple comparison test (black bars). Differences between visits were measured by a Wilcoxon matched-pairs signed ranks test (grey bars). Correlations were measured by Spearman correlation. For single copy VL analysis: n=11 for S1; n=16 for S2; n=11 for S3; and n=5 for S4/5. For plasma neutralization: S3: n=13 for W0/W24, n=12 for W48; S4/5: n=9 for all timepoints. For ADCC and antibody binding measures: S1: n=12 for W0/24, n=11 for W48; S2: n=17 for W0/24, n=15 for W48; S3: n=14 for W0/24, n=13 for W48; and S4/5: n=9 for all timepoints. CHI: n = 4 for untreated and n = 8 for treated. * $p<0.05$; ** $p<0.01$; *** $p<0.001$

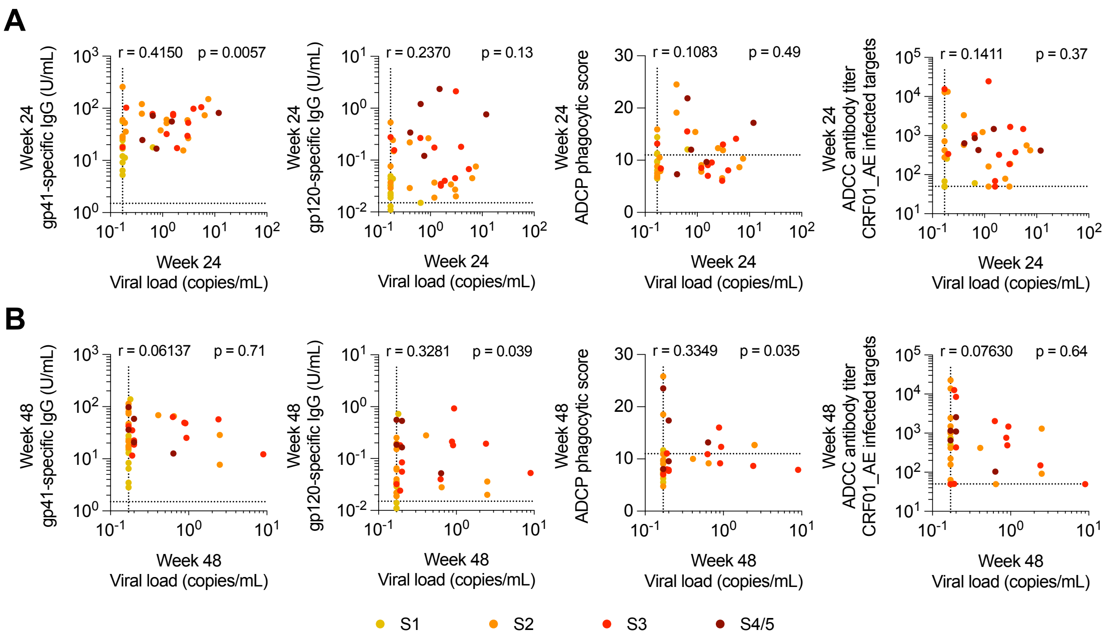

Supplemental Figure 2. Correlations between single copy viral load and antibody levels. Correlations between single copy VLs at week 24 **(A)** or week 48 **(B)** after ART initiation and gp41-specific Abs, gp120-specific Abs, ADCP phagocytic score, or CRF01_AE-specific ADCC titers. Correlations were measured by Spearman correlation. For W24: n=11 for S1; n=16 for S2; n=11 for S3; and n=5 for S4/5. For W48: n=10 for S1; n=14 for S2; n=11 for S3; and n=5 for S4/5

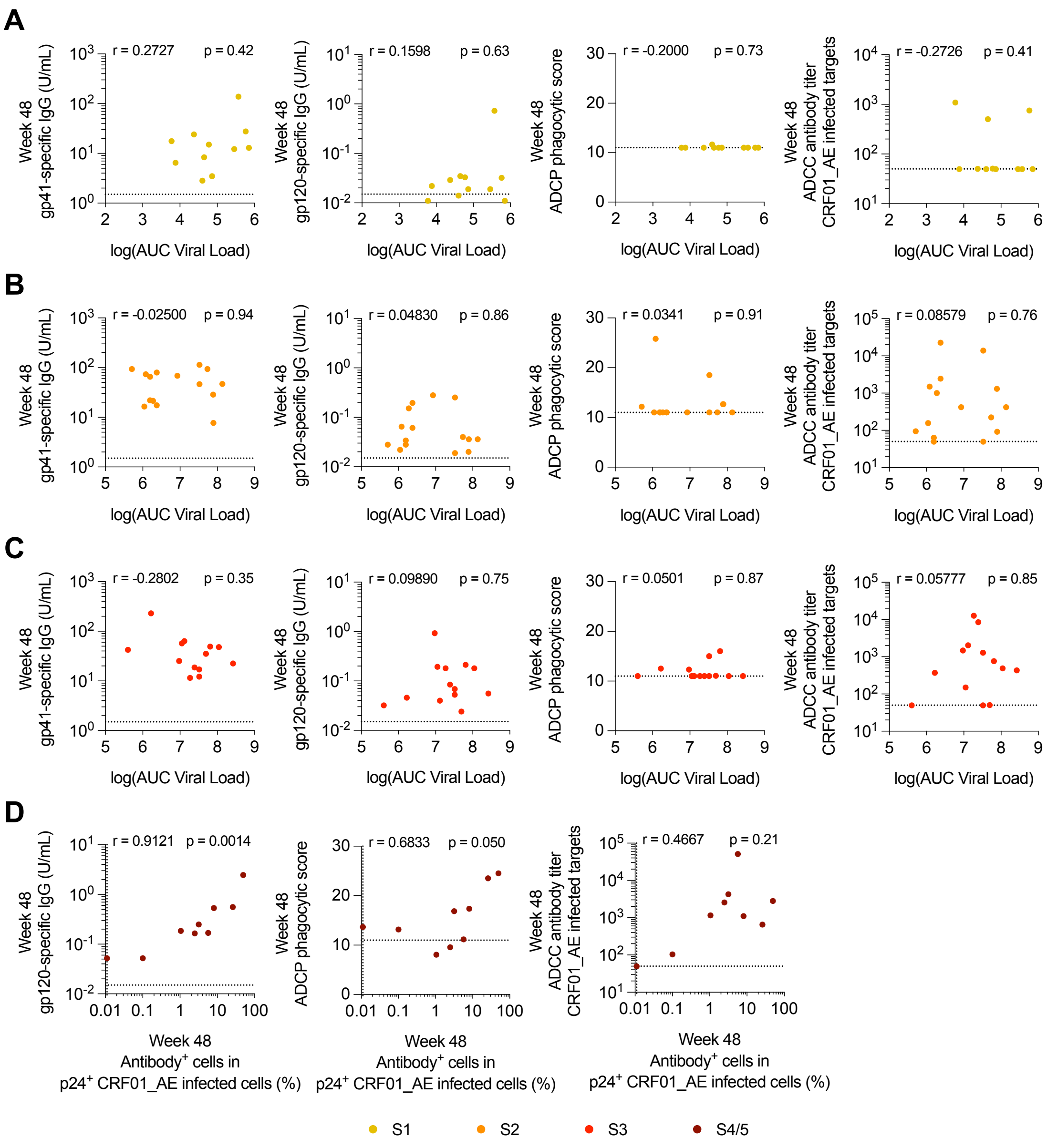

Supplemental Figure 3. Correlations between AUC viral load and antibody levels. Correlations between AUC VL and levels of gp41-specific Abs, gp120-specific Abs, ADCP phagocytic score, and CRF01_AE-specific ADCC titers at W48 after ART initiation in participants who initiated ART in S1 **(A)**, S2 **(B)**, and S3 **(C)** of AHI. **(D)** Correlations between antibody binding activity against CRF01_AE infected cells and the levels of gp120-specific Abs, ADCP phagocytic score, or CRF01_AE-specific ADCC titers at W48 after ART. Correlations were measured by Spearman correlation. n=11 for S1; n=15 for S2; n=13 for S3; and n=9 for S4/5.

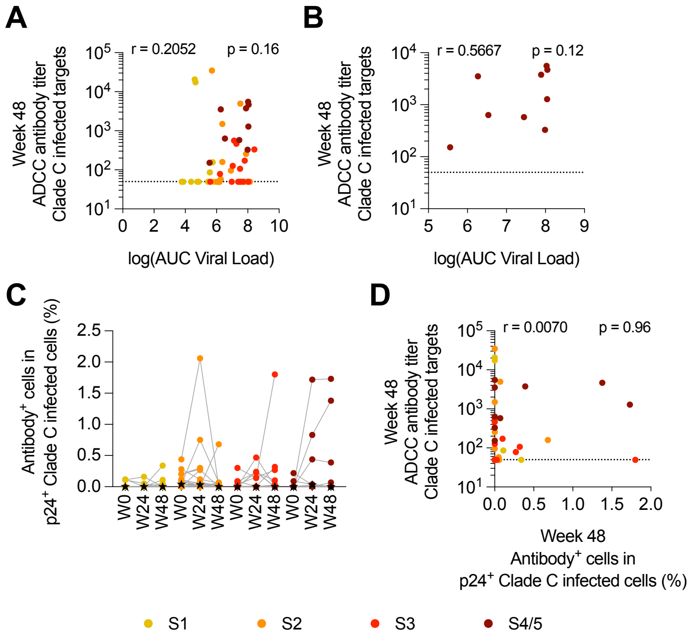

Supplemental Figure 4. Clade C ADCC antibody titer correlations. Correlations between AUC VL and Clade C-specific ADCC titer at W48 after ART initiation are shown for all participants together **(A)** and for those who initiated ART in S4/5 of AHI **(B)**. **(C)** Antibody binding activity against Clade C infected cells was measured using plasma collected at ART initiation or after W24 and W48 of ART. **(D)** Correlation between the level of Clade C-specific antibody binding activity and ADCC antibody titers after 48 weeks of ART. Correlations were measured by Spearman correlation. S1: n=12 for W0/24, n=11 for W48; S2: n=17 for W0/24, n=15 for W48; S3: n=14 for W0/24, n=13 for W48; and S4/5: n=9 for all timepoints.

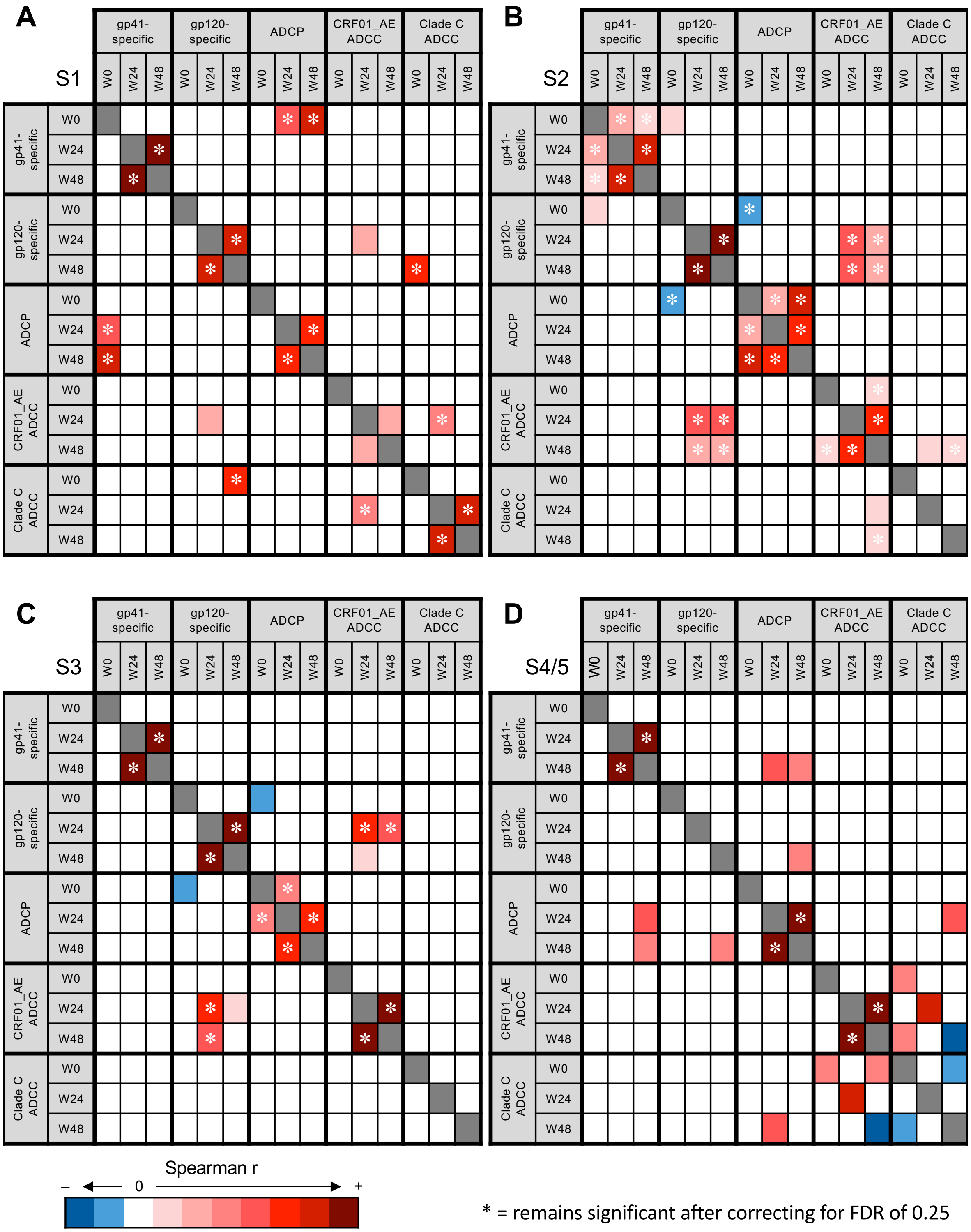

Supplemental Figure 5. Correlations between longitudinal antibody measures by AHI stage when ART was initiated. Correlations were measured between levels of gp41-specific Abs, gp120-specific Abs, ADCP, CRF01_AE ADCC, and Clade C ADCC titers at the three time

points studied (W0, W24, and W48) in participants treated in S1 **(A)**, S2 **(B)**, S3 **(C)**, and S4/5 **(D)** of AHI. Correlations were measured by Spearman correlation. Colored boxes indicate correlations that were significant ($p<0.05$) and are shaded according to Spearman r value, with dark red corresponding to higher r values and dark blue with more negative r values. Asterisks indicate correlations that remained significant after correction for a false discovery rate of 25%. For W0 and W24: n=12 for S1; n=17 for S2; n=14 for S3; and n=9 for S4/5. For W48: n=11 for S1; n=15 for S2; n=13 for S3; and n=9 for S4/5.
